# Site-Directed Mutations and the Polymorphic Variant Ala160Thr in the Human Thromboxane Receptor Uncover a Structural Role for Transmembrane Helix 4

**DOI:** 10.1371/journal.pone.0029996

**Published:** 2012-01-17

**Authors:** Raja Chakraborty, Sai Prasad Pydi, Scott Gleim, Shyamala Dakshinamurti, John Hwa, Prashen Chelikani

**Affiliations:** 1 Department of Oral Biology, University of Manitoba, Winnipeg, Manitoba, Canada; 2 Section of Cardiology, Department of Internal Medicine, School of Medicine, Yale University, New Haven, Connecticut, United States of America; 3 Departments of Pediatrics and Physiology, University of Manitoba, Winnipeg, Manitoba, Canada; 4 Manitoba Institute of Child Health, Winnipeg, Manitoba, Canada; University of Oldenburg, Germany

## Abstract

The human thromboxane A2 receptor (TP), belongs to the prostanoid subfamily of Class A GPCRs and mediates vasoconstriction and promotes thrombosis on binding to thromboxane (TXA2). In Class A GPCRs, transmembrane (TM) helix 4 appears to be a hot spot for non-synonymous single nucleotide polymorphic (nsSNP) variants. Interestingly, A160T is a novel nsSNP variant with unknown structure and function. Additionally, within this helix in TP, Ala160^4.53^ is highly conserved as is Gly164^4.57^. Here we target Ala160^4.53^ and Gly164^4.57^ in the TP for detailed structure-function analysis. Amino acid replacements with smaller residues, A160S and G164A mutants, were tolerated, while bulkier beta-branched replacements, A160T and A160V showed a significant decrease in receptor expression (Bmax). The nsSNP variant A160T displayed significant agonist-independent activity (constitutive activity). Guided by molecular modeling, a series of compensatory mutations were made on TM3, in order to accommodate the bulkier replacements on TM4. The A160V/F115A double mutant showed a moderate increase in expression level compared to either A160V or F115A single mutants. Thermal activity assays showed decrease in receptor stability in the order, wild type>A160S>A160V>A160T>G164A, with G164A being the least stable. Our study reveals that Ala160^4.53^ and Gly164^4.57^ in the TP play critical structural roles in packing of TM3 and TM4 helices. Naturally occurring mutations in conjunction with site-directed replacements can serve as powerful tools in assessing the importance of regional helix-helix interactions.

## Introduction

Membrane receptors present on the cell surface mediate the communication between the cell and its environment. The largest group of these membrane receptors belong to the family of G-protein coupled receptors (GPCRs) [1]. GPCRs contain seven transmembrane helices (TM) and are activated by diverse extracellular stimuli including hormones, tastants, light, peptides and neurotransmitters. The majority of GPCRs belong to the Class A family and are important pharmacological targets, with 40–50% of prescription drugs targeting these receptors. The human thromboxane A2 receptor (TP) belongs to the prostanoid subfamily of Class A GPCRs, and is primarily activated by the prostanoid, thromboxane A_2_ (TXA_2_). TP mediates thrombosis and vasoconstriction, thereby playing an important pathophysiological role in heart disease and stroke [2], [3].

The receptor knockout studies have implicated TXA_2_ as a key regulator of atherosclerosis. Surprisingly little is known about the influence of TP gene (*TBXA2R*) variations on cardiovascular disease. With TXA_2_ being a powerful airway constrictor, the major research has focused on the association of variants with severity and susceptibility to asthma [4],[5] [6],[7] and atopic dermatitis [8], [9]. Structural studies on TP are very limited and few have addressed the functional significance of the polymorphic variants in this receptor [10], [11], [12], [13]. A point mutation (R60L) in the first cytoplasmic loop of the TP, however, has been identified in an autosomal dominant bleeding disorder characterized by defective platelet response [14] and another mutation D304N was also associated with bleeding [13]. None of these studies have provided the details for structural perturbations or evidence for constitutive activity as outlined in our study.

Recent crystal structures and mutational studies of rhodopsin and the β_2_-AR show that TM1-TM4 form a helical bundle core, with other helices moving around this core upon activation [15], [16], [17]. Homo- and heterodimerization studies on GPCRs have shown that TM4 is an important part of the dimer interface [18], [19]. TM4 is one of the shortest helices in GPCRs, yet it performs important structural and functional roles and is a hot spot for naturally occurring GPCR variants. More than 700 GPCRs are identified in the human genome, with a substantial number of these harbouring genetic variants [20], including nucleotide insertion or deletion as well as single nucleotide changes referred to as single nucleotide polymorphisms (SNP). SNPs are sequence changes that can result in either synonymous (i.e. change in DNA sequence but no change in amino acid sequence) or non-synonymous (change in DNA and amino acid sequence) mutations.

Mutations that affect protein structure and function tend to occur at evolutionarily conserved sites and are usually buried in protein structure [21]. One such region in Class A GPCRs, is the TM4 from residues 4.53 to 4.57 (numbering according to Ballesteros and Weinstein nomenclature [22]). For example, in rhodopsin the non-synonymous (ns) SNP A164V^4.53^ destabilizes helix packing resulting in protein misfolding and causes retinitis pigmentosa [23], similarly the T164I^4.56^ nsSNP in β_2_-AR is hypofunctional and is associated with coronary and peripheral artery disease [24]. In the TP receptor, currently there are 7 ns SNPs listed in the GPCR natural variant database [20], a G/A change at position 478 in the nucleotide sequence causes a codon change from GCG>ACG, resulting in A160T in TM4.

**Figure 1:**
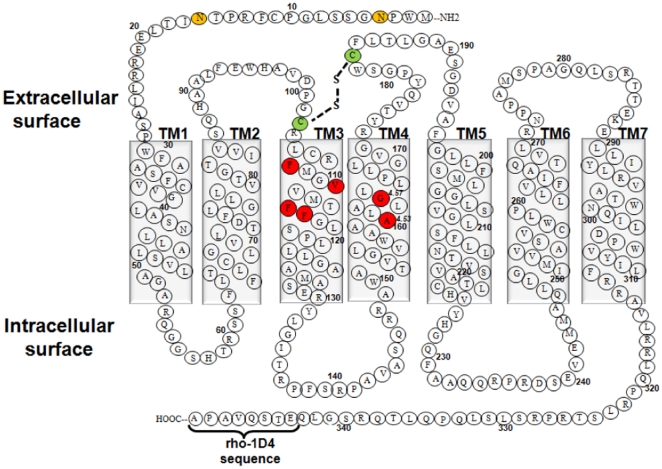
Two-dimensional representation of the TPα amino acid sequence. Amino acids are shown in single-letter codes. Shown are the seven transmembrane helices, the disulphide bond between the Cys102 and Cys183 (green colored residues), the N-glycosylated residues Asn4 and Asn16 (orange colored residues), and the rho-1D4 octapeptide epitope tag at the C-terminus. The two conserved residues Ala160^4.53^ and Gly164^4.57^ on TM4 along with the residues on TM3 mutated in this study are shown in red.

In this manuscript, we target two amino acids present in TM4 of TP receptor, Ala160^4.53^ and Gly164^4.57^ for structure-function analysis (Figure 1). These residues are of particular interest for multiple reasons, the region is highly conserved in Class A GPCRs (categorized as group-conserved residues) [17], [25], with conservation of up to 99% when considered as a group of small and weakly polar residues (Gly, Ala or Ser). These amino acids have been identified in membrane proteins as key determinants in helix-helix interactions [26]. Furthermore, A160T is a novel nsSNP in TP that is yet to be characterized and Gly164 is present in close proximity, just one helical turn away and towards the extracellular surface. In this study, mutants were generated by site-directed mutagenesis and transiently expressed in COS-1 or HEK293T cells, and ligand binding assays were performed using membrane preparations. To elucidate the effect of these mutations on G-protein signaling, changes in intracellular calcium levels were measured following stimulation by agonist U46619. Agonist-independent signaling was also measured to assess constitutive activity. Guided by molecular modeling, a series of compensatory mutations were made on TM3, in order to accommodate the bulkier replacements (example, replacement with valine) at positions 4.53 and 4.57 on TM4. Our results show that Ala160^4.53^ and Gly164^4.57^ in TP receptor form a packing motif and have a structural role in the tight packing of helices TM4 and TM3. The A160V/F115A double mutant showed a moderate increase in expression level compared to either A160V or F115A single mutants. The nsSNP variant A160T decreased receptor stability and demonstrated increased affinity towards the agonist U46619. Both the A160T and A160V mutants displayed agonist-independent signaling. Furthermore, molecular modeling analysis suggested that the G164V mutation at the extracellular end of TM4 causes loss of hydrogen bonding contact of Ser191 in the extracellular loop-2 (ECL2) with antagonist SQ 29,548. Previous studies have shown that the residues on ECL2 in TP receptor are important for SQ 29,548 binding [11], [27].

## Materials and Methods

### Materials

The detergent n-dodecyl-b-D-maltoside (DM) was purchased from Anatrace (Maumee, OH, USA). Fetal Bovine Serum and Dulbecco's Modified Eagle Medium high glucose were purchased from Sigma and Invitrogen. The TP antagonist [^3^H] SQ 29,548 was purchased from PerkinElmer (NET936250UC, PerkinElmer, MA, USA). Unlabelled TP antagonist SQ 29548, and agonist U46619 was purchased from Cayman Chemicals Company (Michigan, USA). Protease inhibitors and common chemicals were purchased either from Fisher or Sigma. Buffers used were as follows: phosphate-buffered saline (PBS) buffer: 137 mM NaCl, 2.7 mM KCl, 1.8 mM KH_2_PO_4_, and 10 mM Na_2_HPO_4_ (pH 7.4); buffer A (lysis buffer), 10 mM Tris-HCl, pH 7.4, containing protease inhibitors (1 mM ethylenediaminetetraacetic acid (EDTA), 10 µg/ml benzamidine, 10 µg/ml leupeptin, 20 µg/ml soybean trypsin inhibitor, 5 µg/ml aprotinin, and 0.2 mM phenylmethylsulfonyl fluoride); buffer B (storage buffer) 50 mM Tris-HCl, pH 7.4, 12.5 mM MgCl2, and containing protease inhibitors as in buffer A; buffer C (binding buffer), 75 mM Tris-HCl, pH 7.4, 12.5 mM MgCl2, and containing protease inhibitors as in buffer A; buffer D, 20 mM Tris-HCl, pH 7.4, containing 100 mM NaCl, and 1 mM EDTA.

### Molecular Biology and Cell culture

Amino acid substitutions were introduced into the synthetic TPα gene carried by the expression vector pMT4 as described previously [28], [29]. DNA sequences of all the mutated genes were verified by automated DNA sequencing. To minimize variations in transfection efficiency, the total amount transfected DNA was kept constant in all cases at 1 µg of DNA per 7×10^5^ cells. The wild type *TP* or mutant genes were expressed in COS-1 cells using a DEAE-dextran based transient transfection method [28], [29], and in HEK293T cells using lipofectamine 2000 (Invitrogen). Except unless specified, the membranes prepared from COS-1 cells were used for radioligand binding assays and immunoblots. For intracellular calcium determination assays and immunofluorescence imaging, HEK293T cells were used. For transient transfections of HEK293T cells using the plasmid pMT4, lipofectamine 2000 (Invitrogen) mediated transfection was used as described by the manufacturer. Membranes were prepared using Buffers A and B and as described previously [28], [29]. The protein concentration in the resuspended membrane pellet was determined using a modified DC protein assay kit from Bio-Rad Laboratories (Hercules, CA).

### Radioligand binding assays

These were carried out in Buffer C for 60 min at room temperature, using 2 to 20 µg of membrane protein and different concentrations of [^3^H] SQ 29,548 (0.5 nM to 20 nM). Binding of [^3^H] SQ 29,548 in the presence of 10 µM SQ 29,548 was used as a measure of nonspecific binding. Competition binding assays were performed using 4 nM [^3^H] SQ 29,548 and different concentrations of unlabeled agonists (10^−3^–10^−9^ M) and the reactions kept for 2 hr at room temperature. Binding was terminated by filtering under vacuum on GF/A filters (Millipore). Filter-bound radioactivity was measured using a liquid scintillation counter. Equilibrium dissociation constants (K_d_) were determined from saturation isotherms using PRISM software version 5.0 (GraphPad Software Inc, San Diego, CA, USA). The K_i_ values were calculated from the IC_50_, using the equation of Cheng and Prusoff by PRISM software version 5.0. Where applicable, statistical significance of the data was evaluated using analysis of variance (ANOVA) and/or unpaired *t* test.

### Determination of intracellular calcium

Changes in intracellular calcium were measured by using the fluorescent calcium sensitive dye Fluo-4NW (Invitrogen). After 6–8 hours of transient transfection of HEK293T cells using lipofectamine 2000, 100,000 viable cells were plated into each well of a 96-well tissue culture treated BD-falcon optilux plates. Cells mock transfected with vector pMT4 alone were used as a negative control. Following 24 hours of incubation at 37°C, the media was removed and cells were incubated with the dye Fluo-4NW (Invitrogen) containing 77 µg/ml of probenecid for 1 hour, as recommended by the manufacturer. Receptor activation was determined by measuring changes in intracellular calcium after application of different concentrations of agonist U46619 for TP and mutants, using Flexstation-3 fluorescence plate reader (Molecular Devices, CA, USA) at 525 nm following excitation at 494 nm. Dose–response curves were generated and EC_50_ values calculated by nonlinear regression analysis using PRISM software version 5.0 (GraphPad Software Inc, San Diego, CA) after subtracting the responses of mock-transfected cells stimulated with same concentrations of agonists.

For estimation of calcium mobilized using the non-ratiometric calcium indicator Fluo-4NW, the ΔF/F ratio which approximately indicates calcium is calculated using the equation (19), ΔF/F =  (F-F_base_)/(F_base_ -B). Where F is the measured fluorescence intensity of Fluo-4NW, F_base_ is the fluorescence intensity of Fluo-4NW in the cell before stimulation, and B is the background signal determined from areas adjacent to the cell. For determination of basal Ca^2+^ levels for agonist-independent signaling, the Ca^2+^ mobilized (ΔF/F) was corrected for receptor expression levels (Bmax in picomoles).

### Thermal sensitivity assays

Aliquots containing membranes of TP or mutant receptors were incubated at 25°C, 37°C and at 42°C for 1 to 5 hrs in buffer C. At the specified time point, aliquots containing the membranes were removed from the water bath and the receptors were incubated with a single saturating concentration (20 nM) of [^3^H] SQ 29,548 for 60 minutes at room temperature and binding was terminated by filtering under vacuum on GF/A filters (Millipore). Filter bound radioactivity was measured using a scintillation counter. Binding of [^3^H] SQ 29,548 in the presence of 10 µM SQ 29,548 was used as a measure of nonspecific binding. The activity of the receptor at starting time point (zero) is taken as 100% for the wild type and respective mutants, and the activity remaining at different time points is expressed as a percentage of the starting time point.

### Immunofluorescence microscopy

HEK293T cells were seeded into six-well tissue culture plates containing sterilized poly-L-lysine (Sigma)-coated glass cover-slips and transiently transfected with TP or the mutant constructs according to published procedures [30]. Cells were fixed in 3.7% paraformaldehyde/1× PBS buffer for 15 min, then permeablized with 0.05% triton X-100/1× PBS buffer for 30 min. The cells were washed and blocked with 1× PBS buffer containing 2% bovine serum albumin (IgG and Protease free) for 60 min. Briefly, TP and the mutants were labelled for 90 min using a 1∶500 dilution of the mouse-anti-rho-1D4 monoclonal antibody (C-terminal tagged TP) and a 1∶100 dilution of rabbit anti-calnexin polyclonal antibody (Abcam, MA, USA; endoplasmic reticulum marker). The transfected cells were washed and incubated with fluorophore-conjugated secondary antibodies using a 1∶2000 dilution of goat anti-mouse Alexafluor 488 (Invitrogen) and 1∶300 dilution of goat anti-rabbit Alexafluor 594 (Invitrogen) for 60 min. Prolong-antifade gold (Invitrogen, Molecular probes, CA, USA) was used to mount the coverslips on slides, and the edges sealed with nail-polish. Representative cells were selected and visualized using an Olympus BX81 microscope for cytoplasmic or plasma membrane localization.

### Homology modeling

The basal model of the TPα was built by homology modeling using the crystal structure of β_1_AR (PDB ID, 2VT4) as template. The transmembrane regions of TP were modelled using MODELLER 9V7 [31]. Loops of the receptors were modelled using loop database of SPDBV4.0.1 [32] based on the available 2D NMR structures of loop regions [33]. Side chains of the molecules were refined with SCWRL4 database [34]. The whole molecule was energy minimized by 1000 steps of steepest descent (SD) and 1000 steps of conjugate gradients by using SPDBV 4.0.1 [32]. Molecular dynamics (MD) simulations were performed for the basal model with OpenMM Zephyr [35]. The quality of model was verified using PROCHEK [36]. This model was used for further mutational and docking studies. The mutants were built using PyMol, and these models were further simulated with OpenMM Zephyr.

The model was then docked with either antagonist SQ 29,548 or agonist U46619 using AUTODOCK VINA [37]. The binding site of ligand on the receptor was defined by forming a cube with dimensions 60×80×70 around the protein with a grid point spacing 0.375 Å and center grid boxes −51.807, −12.467 and 38.921 in X, Y and Z dimensions respectively. We performed 50 runs for each ligand. In each run the best pose or energy minimized conformation was saved. Finally, all poses were superimposed and the most frequent orientation of the ligand was taken as final pose. Receptor ligand complex was further simulated using Desmond 2.4.2.1 molecular dynamics simulation software [38].

## Results

The role of TM4 in the thromboxane receptor and prostanoid receptors in general is unclear. We used a combination of naturally occurring and site-directed mutations, and molecular modelling studies to determine the critical role for this highly conserved region in TP. In humans, TP exists as two isoforms TPα and TPβ which are splice variants of a single gene [39]. These variants differ only in their intracellular carboxyl terminal regions. As the current study is focused on residues involved in helix packing and that are conserved in both the isoforms, the shorter isoform TPα was used. The conserved residues Ala160^4.53^ and Gly164^4.57^ are present on the inward-facing side of TM4 in TP. Elucidating the role of Ala160^4.53^, may also decipher the mechanism of the nsSNP variant A160T. Our initial strategy was to replace Ala160^4.53^ and Gly164^4.57^ by amino acids containing both small and large molecular volumes and to study the effect of these replacements upon receptor expression, activity, and binding of the antagonist SQ 29,548.

### Expression and ligand binding properties of TP and mutants

**Figure 2:**
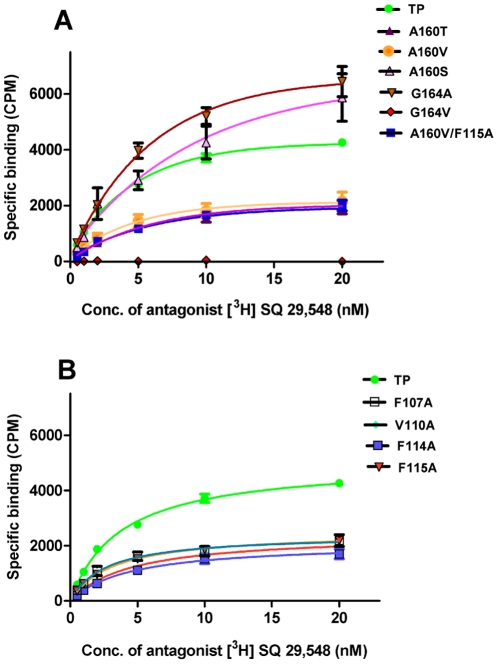
Saturation binding assays of wild type TP and the mutant receptors using the TP antagonist [^3^H] SQ 29,548. Saturation assays with membrane bound TP and the mutant receptors, TM4 mutants in panel A and TM3 mutants in panel B, were performed with different concentrations of [^3^H] SQ 29,548. A one way ANOVA analysis without any post hoc test showed that except for G164V in TM4 there were no significant difference between TP and the mutants at significance level of p<0.05. The data is from a minimum of three independent experiments, with each point in duplicate.

The ligand binding properties of the TP mutants were measured using the antagonist SQ 29,548 (Table 1). Conservative substitutions of Ala160^4.53^ and Gly164^4.57^ with small amino acids were better tolerated with the A160S and G164A mutants showing an increase in expression of functional receptor as quantified by B_max_ values (Table 1), and saturation isotherms in Figure 2. A one way ANOVA analysis without any post hoc test showed that except for G164V in TM4 there were no significant difference between TP and the mutants at significance level of p<0.05. Replacement with larger amino acids at position Ala160^4.53^ caused significant reduction in receptor expression (Figure 2). Gly164^4.57^ was more sensitive with the G164V mutant losing the ability to bind to the antagonist SQ 29,548 (Figure 2). To verify whether this loss is due to protein misfolding, G164V immunofluorescence microscopy showed that the receptor was properly expressed on the cell surface (Figure S1). The A160T and A160V mutants with bulky β-branched amino acids were expressed at half of the levels of wild type as reflected by the lower B_max_ values (Table 1). This is in agreement with previous studies on TM4 residues, Ser161^4.53^ and Ser165^4.57^ of β_2_-AR, where it was observed that conservative substitutions with small amino acids such as alanine to serine had a minimal effect on receptor folding and ligand binding, whereas mutations made to a non-group conserved amino acid with bulky side chains, such as alanine or serine to valine and leucine, reduced receptor expression [17], [40].

**Table 1:** Summary of ligand binding properties of wild type TP and mutants*.

Receptor	Transmembrane helix	K_d_ 1 (nM)	95% confidence intervals	B_max_ 2(pmol/mg)	EC_50_ 3(nM)
WT-TP		3.7	3.02 to 4.44	3.8±0.3	13.1±0.8
A160S^4.53^	IV	6.3	3.64 to 8.90	5.8±0.5	9.3±0.3
A160T	IV	4.8	3.17 to 6.42	1.8±0.4	5.5±0.3
A160V	IV	3.8	1.08 to 6.47	1.9±0.3	9.5±0.2
G164A^4.57^	IV	5.9	3.88 to 7.99	6.3±0.4	2.5±0.2
G164V┼	IV	-			38.0±2.1
F107A^3.27^	III	2.6	1.33 to 3.92	1.8±0.2	2.5±0.7
V110A^3.30^	III	4.6	3.72 to 5.44	1.8±0.3	2.7±0.2
F114A^3.34^	III	4.5	2.14 to 6.84	1.6±0.3	1.8±0.2
F115A^3.35^	III	3.2	2.50 to 3.89	1.8±0.2	2.0±0.3
A160V/F114A┼	IV/III	-			
G164V/F107A┼	IV/III	-			
G164V/V110A┼	IV/III	-			
A160V/F115A	IV/III	5.0	1.83 to 8.13	2.1±0.4	43.5±1.5

*The values are expressed as the mean ± S.E (n = 2 to 5 experiments in duplicate), and the experiments are performed using the TP antagonist [^3^H] SQ 29,548 as the radioligand (NET936250UC, PerklinElmer).

┼ No significant specific binding to [^3^H] SQ 29,548 detected for these mutant receptors under the assay conditions.

1 K_d_, Affinity of the antagonist SQ 29,548 for the receptor.

2 B_max_, Binding maximum of the ligand SQ 29,548 for the receptor. Usually expressed as picomoles of TP per milligram of total membrane protein.

3 EC_50_ the molar concentration of agonist U46619 that produces 50% of the maximal possible effect (calcium mobilized) for TP and mutant receptors.

Substitution of the two residues Ala160^4.53^ and Gly164^4.57^, on TM4 with large amino acids may affect the proper packing of the helices due to steric interactions. If this is the case, introduction of a second mutation at an appropriate site on an opposing helix may compensate for the steric clash and restore correct packing. Using the ligand-free TP molecular model as a template, possible compensatory mutants were designed by selecting residues within 5 Å of Ala160^4.53^ and Gly164^4.57^, for mutagenesis. In the ligand-free TP model, Ala160^4.53^ is close to Phe114^3.34^ and Phe115^3.35^, whereas Phe107^3.27^ and Val110^3.30^ are in proximity of Gly164^4.57^. Based upon molecular modeling, a series of compensatory mutations F107A, V110A, F114A and F115A were made on TM3, in order to try to accommodate the bulkier residues (Table 1). The single mutants on TM3 bound to antagonist with affinities similar to TP (Table 1). Additionally they showed decreased expression levels probably because the large molecular volumes of the amino acids replaced could not be compensated for by the smaller volumes which might have affected the local hydrophobic environment. All the compensatory double mutations except for A160V/F115A lacked the ability to bind to the antagonist. The A160V/F115A double mutant showed a moderate increase in expression level compared to either A160V or F115A single mutants. To elucidate whether the double mutants were misfolded and/or unable to bind to the antagonist, immunofluorescence microscopy was performed and A160V/F115A, G164V/V110A mutants were found localized on the cell surface. A160V/F114A and G164V/F107A double mutants were predominantly retained in the intracellular compartments (Figure S1). This result shows that the G164V/V110A mutant was properly trafficked to the cell surface but was unable to bind to the antagonist, resembling the G164V phenotype.

### Agonist competition binding assays

**Figure 3:**
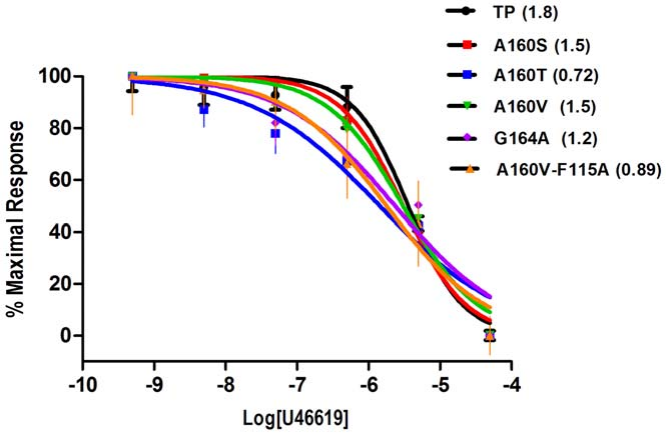
Heterologous competition curves of TP and select mutants using the unlabeled agonist U46619 and antagonist [^3^H] SQ 29,548. The Ki values (µM) are shown in parenthesis.

We carried out competition radioligand binding assays using the unlabeled agonist U46619 and antagonist [^3^H] SQ 29,548 on TM4 mutants. Results from the heterologous competition assays showed that A160S and A160V mutants displayed moderately increased affinity towards the agonist U46619 with Ki values of 1.52 µM (95% CI, 0.89–2.61) and 1.51 µM (95% CI, 0.89–2.6) compared to 1.80 µM (95% CI, 1.15–2.60) for TP (Figure 3). Significant changes were observed for the A160T and G164A mutants that displayed Ki values of 0.72 µM (95% CI, 0.30–1.66) and 1.17 µM (95% CI, 0.54–2.52). A one way ANOVA showed there is a significant difference between the mutants and TP at significance level of P<0.01 (n = 3). The Ki value of G164V mutant could not be determined because it did not bind to the antagonist under our assay conditions. While Gly164^4.57^ is more than 7 Å from the TP receptor ligand binding pocket, the changes observed for the G164A and G164V mutants can be attributed to the structural influence of amino acid at position 4.57 on the ECL2 (see modeling). The increase in affinity of A160T for the agonist U46619 might be due to indirect effects, i.e., changes in ligand binding pocket or the receptor adopting an active state conformation, due to perturbation of helical packing by the A160T mutant. To elucidate the observed differences in agonist affinities, and decipher whether the mutations affected G protein coupling or activation, measurement of changes in intracellular Ca^2+^ upon agonist stimulation were pursued.

### Intracellular Ca^2+^ signalling

**Figure 4:**
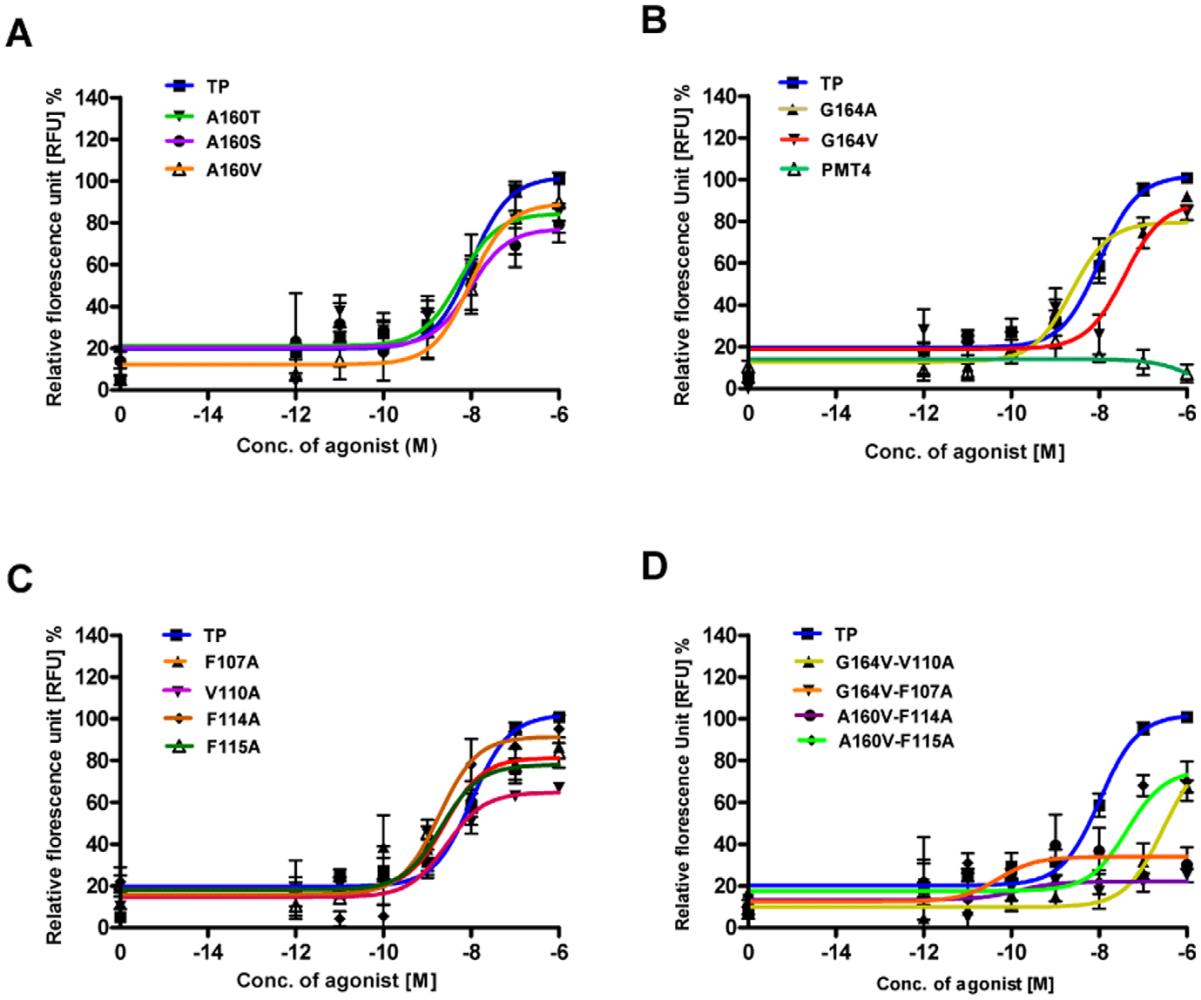
Characterization of Gαq-mediated signalling of the TP and mutant receptors. The data shows agonist U46619 induced calcium release for TP, mutants and mock transfected (vector pMT4) HEK293T cells, and is expressed as a percentage of the TP activity. Ala160 mutants (panel A), Gly164 mutants and mock transfected cells (panel B), TM3 mutants (panel C) and double mutants (panel D). The results are from at least three independent experiments performed in duplicate.

Characterisation of Gαq mediated signaling of the TP receptor and mutants were carried out by measuring the intracellular Ca^2+^ flux upon stimulation with agonist U46619 (Figure 4). No significant variation was found in the EC_50_ values of A160S and A160V compared to wild type (Table 1). In agreement with the agonist competition assays, the A160T mutant showed moderate increased potency towards the agonist U46619, as illustrated by a left shift in the dose response (Figure 4A). Significant changes were seen with the Gly164^4.57^ mutants, the G164A demonstrated increased potency, and G164V mutant showed reduced potency as demonstrated with a right shift in dose response with agonist U46619 (Figure 4B). Nevertheless, the G164V mutant reached up to 90% of wild type activation upon stimulation with higher doses of agonist U46619.

The TM3 mutants F107A, V110A, F114A and F115A upon stimulation with agonist U46619 showed an increase in intracellular Ca^2+^ flux equivalent to 60–80% of TP generated signal (Figure 4C). Interestingly, the TM3 mutants displayed a left shift in the dose response curves, showing that these mutants had increased potency. Indeed, these mutants displayed 4 to 7 fold increase in U46619 potency (decrease in EC_50_ concentration for half-maximum response) with EC_50_ values from 1.8 nM to 2.7 nM compared to EC_50_ of 13 nM for TP (Table 1). Two double mutants, one each at positions 4.53 and 4.57 showed agonist dependent signaling but differed in their dose response characteristics. The double mutant A160V/F115A showed reduced potency as demonstrated by a right shift in dose response (Figure 4D). The G164V/V110A mutant lost SQ 29,548 binding and exhibited a reduced calcium response which could be restored upon stimulation with elevated levels of U46619.

**Figure 5:**
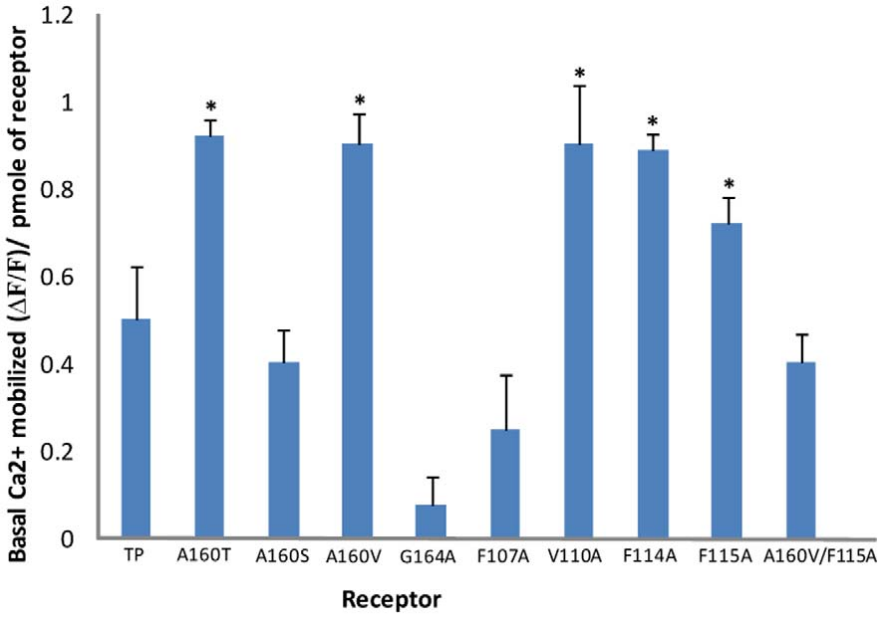
Characterization of basal or agonist-independent activity. Bar plot representation of the basal amount of calcium released by TP and mutants per picomole of functional protein expressed (see methods). The G164V mutant could not be assessed as it did not bind to the antagonist, and the amount of functional receptor could not be calculated. Results are obtained from a minimum of two independent experiments done in duplicate. The single astrix indicate there is a significant difference in the amount of calcium released at zero concentration of agonist with respect to wild type TP at significance level p<0.05. Error bars indicate mean ± SD.

Basal Ca^2+^ levels of the TP mutants corrected for receptor expression levels were measured to assess constitutive activity. Among the TM4 mutants, only bulky β-branched replacements at position 4.53, A160T and A160V displayed a 2-fold increase in agonist-independent activity (Figure 5). The constitutive activity and the reversal of basal activity for the polymorphic variant A160T was characterized (Figure S2). In addition, three TM3 mutants V110A, F114A and F115A displayed constitutive activity (Figure 5). This is not surprising as the mutated residues are towards the extracellular side of TM3 that is known to play a pivotal role in ligand binding and activation in many Class A GPCRs [41], [42].

### Thermal sensitivity assays

**Figure 6:**
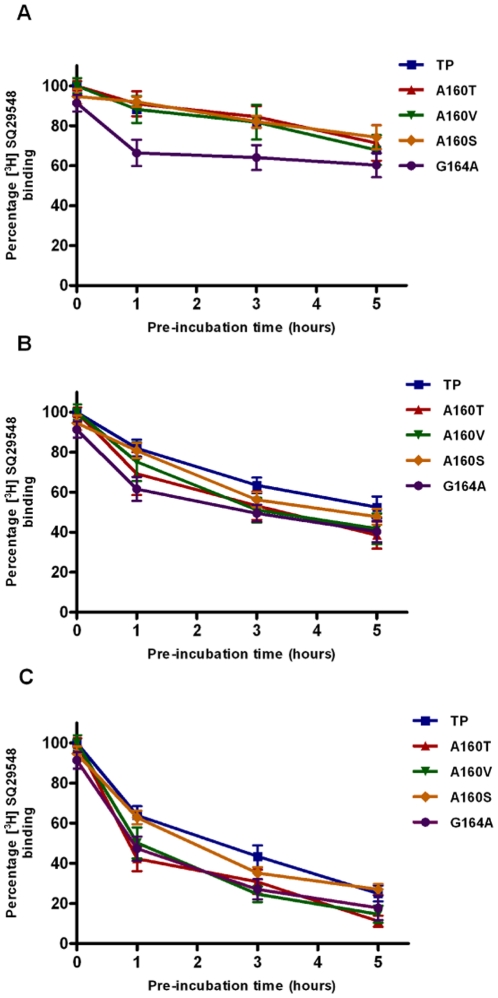
Thermal sensitivity of wild type TP and select mutants. It was measured in terms of the ability of the TP and mutants at positions 4.53 and 4.57 to retain antagonist binding after incubation at 25°C (panel A), 37°C (panel B) and 42°C (panel C) as a function of time. The receptor activity decreased in the order, TP>A160S>A160V>A160T>G164A, with G164A being the least stable. The results are mean ±SE and are from minimum of three independent experiments done in duplicate.

To examine the stability of the TM4 mutants, we monitored the ability of the mutants to retain antagonist affinity after incubation at 25°C, 37°C and 42°C as a function of time (Figure 6). Thermal sensitivity of the wild-type or TM4 mutant receptors was compared to distinguish the contribution of residues at positions 4.53 and 4.57 to stability of the helical core. The wild-type and mutants A160T, A160S and A160V were stable for 5 hrs at 25°C, whereas the G164A mutant showed about a 30–40% loss in antagonist binding (Figure 6A). Thus, differences between the stabilities of different Ala160 mutants could not be readily discerned at 25°C. However, thermal stability differences between the Ala160 mutants were more apparent at higher temperatures of 37°C and 42°C (Figure 6B and C). Within 1 hr at 42°C, mutants A160T and A160V showed 50–60% loss in total binding similar to G164A, while wild type and A160S showed only 30% loss in total binding. Therefore, it appears that the contribution of Gly164 to receptor stability is very important, and replacement by amino acids with small molecular volumes such as alanine (G164A) causing loss of receptor stability. Interestingly, G164A mutant showed close to two-fold increase in expression of functional receptor compared to wild type (Figure 2). This result was surprising as decreased protein stability normally leads to decreased expression, as is the case with A160T and A160V mutants. We speculate that the G164A mutant might be resistant to proteolysis. Data were determined to be statistically significant using the unpaired student *t* test at significance level of p<0.05.

### Molecular modeling

**Figure 7:**
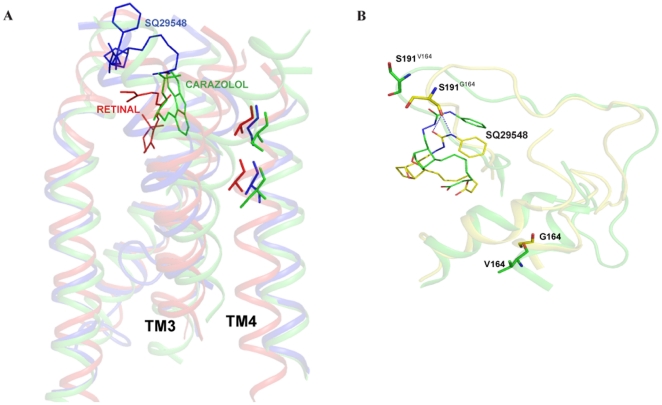
Molecular models of antagonist bound TP and G164V mutant and comparison with the crystal structures of rhodopsin and antagonist bound β_2_-AR. **Panel A**, Molecular model of TP bound SQ 29,548 superimposed with the structures of rhodopsin (PDB ID 1U19) and antagonist bound β_2_-AR (PDB ID 2RH1). The two residues at positions 4.53 and 4.57 on TM4 in both rhodopsin and β_2_-AR were previously studied. For structural comparison all the three structures were superimposed. The color coding is as follows; Ala164 and Ala168 in rhodopsin (red), Ala160 and Gly164 in TP (blue) and Ser161 and Ser164 in β_2_-AR (green). **Panel B**, Molecular models of TP (yellow) and G164V (green) superimposed. The important amino acids Gly164, Val164 and Ser191 are shown. Notice that Ser191 loses interaction with the antagonist SQ 29,548 in the G164V mutant.

Molecular models of TP and the TM4 mutants were constructed to interpret the results in structural terms. Figure 7A shows the homology model of SQ 29,548 bound TP model superimposed with the structures of rhodopsin (PDB ID, 1U19) and antagonist bound β_2_-AR (PDB ID, 2RH1). The SQ 29,548 bound TP model shows very good homology to rhodopsin and β_2_-AR crystal structures with backbone Cα RMSD of 0.4 Å and 1.5 Å for TM4 and TM3 regions analyzed in this study.

**Figure 8:**
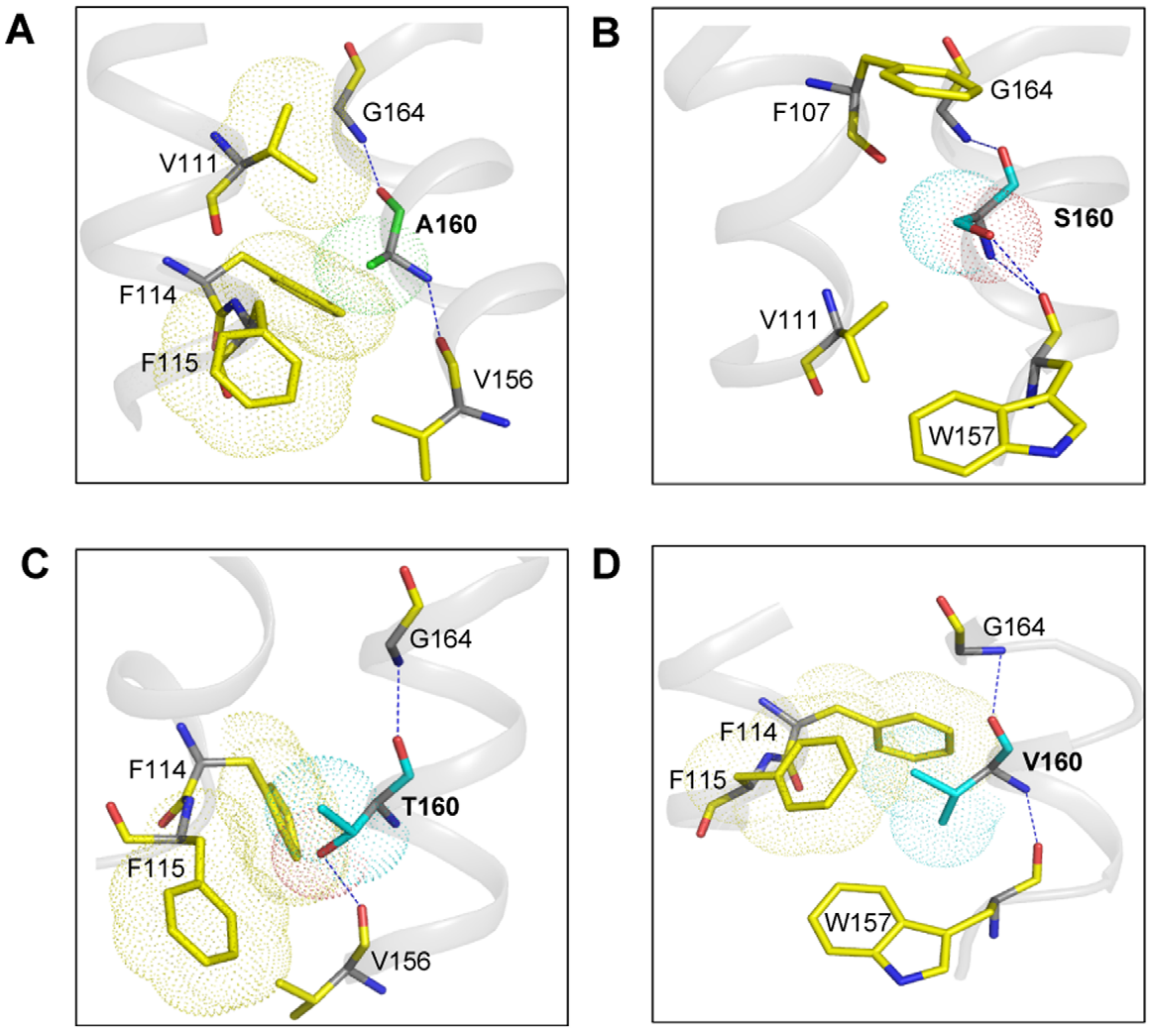
Molecular models of agonist bound TP and Ala160 mutants. Wild type (panel A), A160S (panel B), A160T (panel C) and A160V (panel D), and the important amino acids are shown in each model.

The ECL2 has been shown to play an important role in ligand binding and activation in the TP [11], [27] and other Class A GPCRs [43], [44]. Gly164^4.57^ occupies a crucial position on the extracellular side of TM4, and is located at the base of the N-terminal end of ECL2. In wild type TP and G164A models, Ser191 and Asp193 were found within the 4 Å region of the antagonist molecule SQ 29,548. While in G164V model, Asp193 is within the 4 Å region but the crucial hydrogen bond contact between Ser191 and SQ 29,548 was absent. Mutational studies by Kasawneh *et al.*, [27] have shown that replacement of Ser191 on ECL2 resulted in mutants that were incapable of binding to SQ 29,548 but retained the functional response to treatment with U46619. This is similar to the G164V phenotype we observed, and our models validate this interaction (Figure 7B). In wild type, Ala160^4.53^ is packed between Val111^3.31^, Phe114^3.34^ and Phe115^3.35^. Interestingly, in the A160S mutant the side chain β-OH of Ser160 interacts with the main chain carbonyl oxy-group of Trp157^4.50^ and no steric hindrance from residues on TM4 was observed (Figure 8). In A160V and A160T mutants, the bulky β-branched side chains are intercalated between Phe114^3.34^ and Phe115^3.35^. In agreement with the molecular model, the F115A mutant was able to rescue, albeit partially, the expression of A160V in the A160V/F115A double mutant (Table 1). In our model of the A160T mutant, the Thr160 is more than 8 Å from the ligand binding pocket and is not in a position to directly interact with the ligand (Figure 7 and Figure 8), hence the increase in potency observed is due to its packing interactions with the Phe114^3.34^ and Phe115^3.35^ on TM3, which themselves are very sensitive to replacements and show increased agonist affinity and constitutive activity. Therefore, the agonist-independent activity observed for the A160T and A160V mutants is due to the steric clash with Phe114^3.34^ and Phe115^3.35^ on TM3 resulting in the receptor adopting an active state conformation.

## Discussion

### Role of Ala160^4.53^ and Gly164^4.57^ in TP

The results presented in this report provide important new insights into the role of TM4 in prostanoid receptors and in particular the two conserved residues Ala160^4.53^ and Gly164^4.57^ present in TM4. The residues at positions 4.53 and 4.57 in the TP perform predominantly a structural role in packing of TM3 and TM4 helices. Recently several structures have been reported for agonist-bound Class A GPCRs, which showed that agonist induced conformational changes involves rearrangement of the TM3-TM5-TM6 interface. The recent structural elucidation of a constitutively active rhodopsin mutant, E113Q present on TM3, reinforces the central role of TM3 in GPCR activation [15]. However studies that give insights into the structural requirements for the constitutive and the agonist-induced conformational changes in TM3 among the prostanoid receptors are unavailable.

In the TP, the G164V replacement was unable to bind to the antagonist but showed an agonist dose dependent calcium increase. This phenotype was not observed in rhodopsin and β_2_-AR mutated at the same position (4.57). In the β_2_-AR, compensatory mutants designed to rescue the expression of S161V^4.53^ were unsuccessful but V114A^3.33^/S165V^4.57^ double mutant rescued the expression of S165V^4.57^, while in rhodopsin L119A^3.34^/A164V^4.53^ rescued the defect in chromophore formation caused by the retinitis pigmentosa mutant A164V^4.53^ [17], [23]. However, in the TP we did not observe any protein misfolding upon mutation of either Ala160^4.53^ or Gly164^4.57^ residues. This is in contrast to rhodopsin, where the nsSNP A164V destabilizes helix packing resulting in protein misfolding and retinitis pigmentosa [23]. Interestingly, the human red cone opsin polymorphic variant Ser180Ala (homologous to the rhodopsin nsSNP A164V) accounts for the subtle difference in normal color vision and influences the severity of red-green color vision deficiency [45].

We can speculate as to why the compensatory mutants in TP were not as successful compared to rhodopsin and β_2_-AR. In this study, the compensatory mutations were designed based on proximity between two amino acids in the molecular model, it is possible that there is a network of interhelical hydrophobic interactions involving Ala160^4.53^, Phe114^3.34^and Phe115^3.35^, while subtle changes are tolerated (mutations to smaller amino acids) any major change (replacement with larger residues or double replacements) would disturb this hydrophobic network causing changes to the ligand binding pocket in the TP.

### Polymorphic variant Ala160Thr

Studies have shown that more than 80% of the disease causing mutations affects protein stability [46]. Similar to the decreased thermal stability and constitutive activity observed for the nsSNP A160T, a recent study on the rhodopsin retinitis pigmentosa mutant, G90V, shows that it has low thermal stability in the dark state and is constitutively active [47]. Analysis of the protein sequences revealed that disease causing SNPs tend to occur at conserved sites [48]. The transmembrane region from residues 4.53 to 4.57 in Class A GPCRs, consist of amino acids with small molecular volumes that are highly conserved. While this region is well studied in the opsin and amine subfamilies of Class A GCPRs, and the disease causing nsSNPs characterized, comparable studies in the prostanoid subfamily have not been pursued thus far. Amino acid sequence analyses of 46 TP sequences showed 81% to have alanine at 4.53 and 85% have glycine at position 4.57. The other residues were serine (16%) at 4.53 and alanine (13%) at 4.57, both amino acids with small molecular volumes. Our study reveals a structural role for the nsSNP A160T variant, while the clinical significance of this nsSNP remains to be determined.

In conclusion, we show that the two conserved residues Ala160^4.53^ and Gly164^4.57^ perform a structural role by enhancing interhelical packing, and in stabilizing the inactive conformation of the receptor. A bulky residue at position 4.57 as in the G164V mutant perturbed helical packing causing decreased protein stability but also influenced the interaction between amino acids on ECL2 and the antagonist. This result implies that Gly164^4.57^ indirectly affects ligand binding in TP. Our results reveal the molecular mechanism of the nsSNP variant A160T and show that it has a destabilizing effect on the TP protein structure and causes the receptor to adopt an active state conformation. Together, the results presented above indicate that the study of naturally occurring mutations in conjunction with site-directed mutagenesis can serve as powerful tools in assessing the importance of regional helix-helix interactions in GPCRs and other integral membrane proteins.

## Supporting Information

Figure S1 **Cellular localization of the wild type TP and mutants in HEK293T cells.** Double-label immunofluorescence was performed using mouse monoclonal anti-rho-1D4 antibody which recognizes the C-terminal octapeptide tag on the expressed receptors, and rabbit polyclonal anti-calnexin antibody which localizes to the endoplasmic reticulum (ER). The wild type and mutant receptors were visualized using goat anti-mouse Alexafluor 488 secondary antibody (panel A) and the ER was visualized with goat anti-rabbit Alexafluor 594 secondary antibody (panel B). The overlay of the receptor and ER is shown in panel C (location of the expressed receptor is indicated by an arrow).
(TIF)

Figure S2 **A**. Effect of receptor density on basal Ca^2+^ mobilization. WT-TP (blue line) and A160T (green line) constructs were expressed in HEK293T cells at different receptor densities by varying amounts of DNA used in each transfection (3 µg to 9 µg DNA per 10^5^ cells). Receptor expression levels were determined by radioligand binding assays using a single saturating concentration (20 nM) of [^3^H] SQ 29,548. The slope of WT-TP was 0.002732±0.001595 of calcium mobilized (ΔF/F)/pmol of receptor while that of the A160T was 0.1001±0.04041. **B**. Reversal of basal activation. Agonist-independent calcium mobilization for WT-TP and A160T (grey bars), and calcium release after cells were pre-treated with 1 µM of unlabelled antagonist SQ 29,548 (black bars). Unpaired students *t* test between A160T basal calcium mobilization and A160T pre-treated with SQ 29,548 showed a significant decrease in basal activity at p<0.05. Similar results were obtained for WT-TP. The results are from 2 independent experiments done in triplicate and are represented as Mean ± SD.
(DOC)
