# Supplementary figures and images for: Site-Directed Mutations and the Polymorphic Variant Ala160Thr in the Human Thromboxane Receptor Uncover a Structural Role for Transmembrane Helix 4
Source: PLoS One. 2012 Jan 17;7(1):e29996. doi: 10.1371/journal.pone.0029996 (PMC3260207; doi:10.1371/journal.pone.0029996)

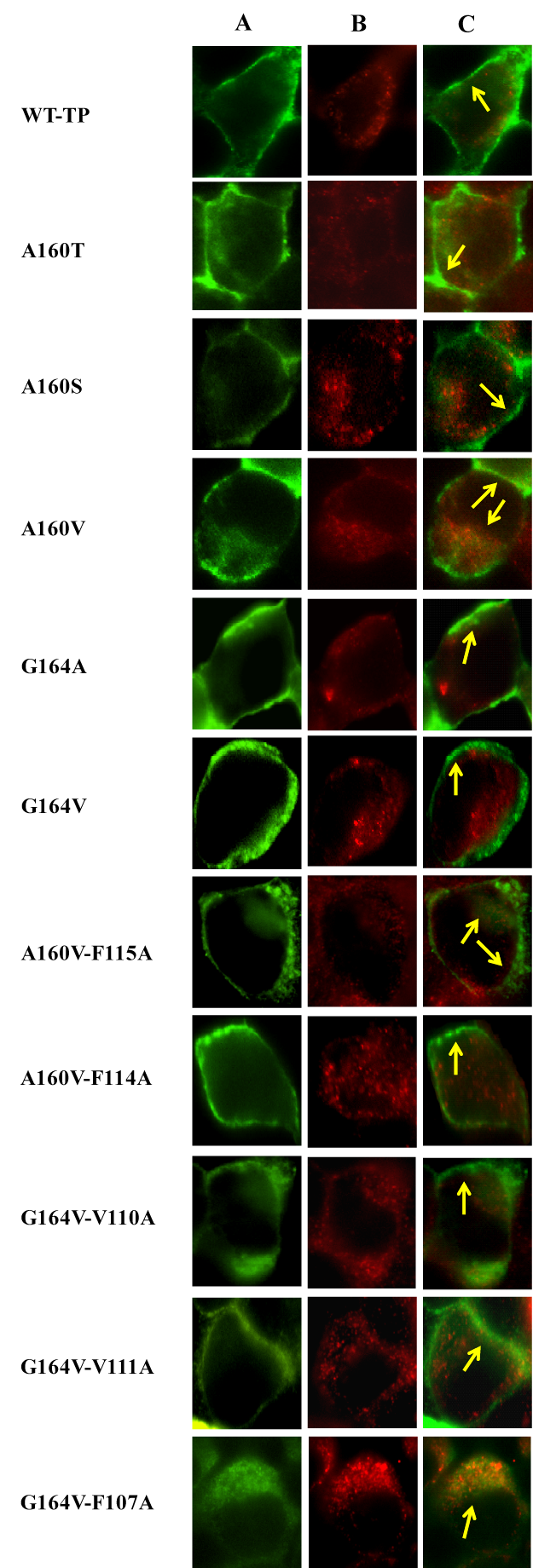

Supplement: Figure S1 — Cellular localization of the wild type TP and mutants in HEK293T cells. Double-label immunofluorescence was performed using mouse monoclonal anti-rho-1D4 antibody which recognizes the C-terminal octapeptide tag on the expressed receptors, and rabbit polyclonal anti-calnexin antibody which localizes to the endoplasmic reticulum (ER). The wild type and mutant receptors were visualized using goat anti-mouse Alexafluor 488 secondary antibody (panel A) and the ER was visualized with goat anti-rabbit Alexafluor 594 secondary antibody (panel B). The overlay of the receptor and ER is shown in panel C (location of the expressed receptor is indicated by an arrow). (TIF) [file pone.0029996.s001.tif]
