# Supplementary material for: Site-Directed Mutations and the Polymorphic Variant Ala160Thr in the Human Thromboxane Receptor Uncover a Structural Role for Transmembrane Helix 4
Source: PLoS One. 2012 Jan 17;7(1):e29996. doi: 10.1371/journal.pone.0029996 (PMC3260207; doi:10.1371/journal.pone.0029996)
Supplement: Figure S2 — A. Effect of receptor density on basal Ca2+ mobilization. WT-TP (blue line) and A160T (green line) constructs were expressed in HEK293T cells at different receptor densities by varying amounts of DNA used in each transfection (3 µg to 9 µg DNA per 105 cells). Receptor expression levels were determined by radioligand binding assays using a single saturating concentration (20 nM) of [3H] SQ 29,548. The slope of WT-TP was 0.002732±0.001595 of calcium mobilized (ΔF/F)/pmol of receptor while that of the A160T was 0.1001±0.04041. B. Reversal of basal activation. Agonist-independent calcium mobilization for WT-TP and A160T (grey bars), and calcium release after cells were pre-treated with 1 µM of unlabelled antagonist SQ 29,548 (black bars). Unpaired students t test between A160T basal calcium mobilization and A160T pre-treated with SQ 29,548 showed a significant decrease in basal activity at p<0.05. Similar results were obtained for WT-TP. The results are from 2 independent experiments done in triplicate and are represented as Mean ± SD. (DOC) [file pone.0029996.s002.doc]

**Figure S2**


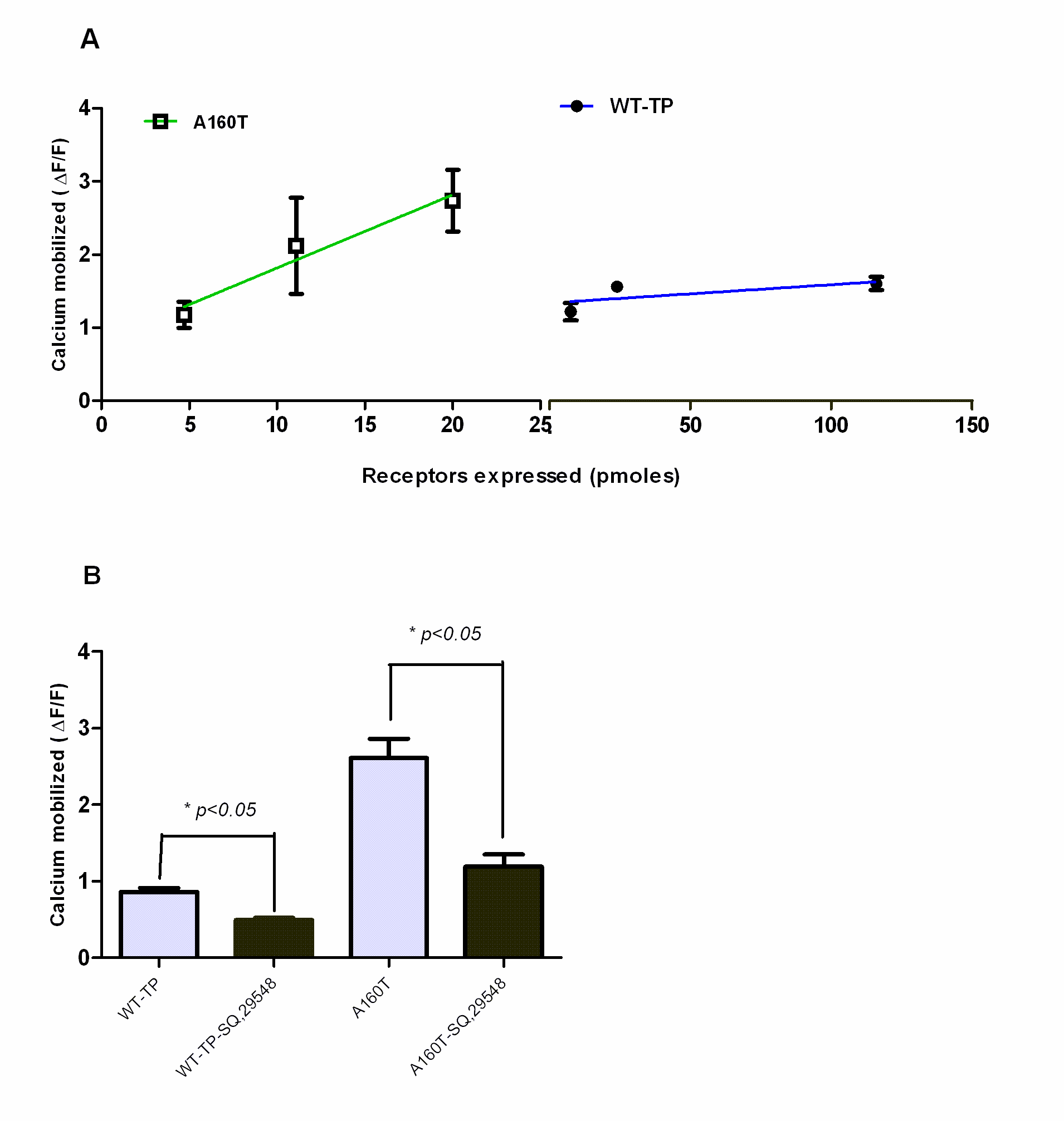


**Figure S2:** **A**. Effect of receptor density on basal Ca^2+^ mobilization. WT-TP (blue line) and A160T (green line) constructs were expressed in HEK293T cells at different receptor densities by varying amounts of DNA used in each transfection (3µg to 9 µg DNA per 10^5^ cells). Receptor expression levels were determined by radioligand binding assays using a single saturating concentration (20nM) of [^3^H] SQ 29,548. The slope of WT-TP was 0.002732 ± 0.001595 of calcium mobilized (ΔF/F) /pmol of receptor while that of the A160T was 0.1001 ± 0.04041. **B**. Reversal of basal activation. Agonist-independent calcium mobilization for WT-TP and A160T (grey bars), and calcium release after cells were pre-treated with 1µM of unlabelled antagonist SQ 29,548 (black bars). Unpaired students *t* test between A160T basal calcium mobilization and A160T pre-treated with SQ 29,548 showed a significant decrease in basal activity at p<0.05. Similar results were obtained for WT-TP. The results are from 2 independent experiments done in triplicate and are represented as Mean + SD.
